# An autoregressive distributed lag approach for estimating the nexus between CO_2_ emissions and economic determinants in Pakistan

**DOI:** 10.1371/journal.pone.0285854

**Published:** 2023-05-25

**Authors:** Muhammad Daniyal, Kassim Tawiah, Moiz Qureshi, Mohammad Haseeb, Killian Asampana Asosega, Mustafa Kamal, Masood ur Rehman

**Affiliations:** 1 Department of Statistics, The Islamia University of Bahawlapur, Bahawlapur, Pakistan; 2 Department of Mathematics and Statistics, University of Energy and Natural Resources, Sunyani, Ghana; 3 Department of Statistics and Actuarial Science, Kwame Nkrumah University of Science and Technology, Kumasi, Ghana; 4 Department of Statistics, Shaheed Benazir Bhutto University, Shaheed Benazirabad Nawabsha, Pakistan; 5 China Institute of Development Strategy and Planning, and Centre for Industrial Economics, Wuhan University, Wuhan, China; 6 Department of Basic Sciences, Saudi Electronic University, Dammam, Saudi Arabia; 7 Department of Information Technology, Saudi Electronic University, Dammam, Saudi Arabia; Institute for Advanced Sustainability Studies, GERMANY

## Abstract

Carbon dioxide (CO_2_) emissions have become a critical aspect of the economic and sustainable development indicators of every country. In Pakistan, where there is a substantial increase in the population, industrialization, and demand for electricity production from different resources, the fear of an increase in CO_2_ emissions cannot be ignored. This study explores the link that betwixt CO_2_ emissions with different significant economic indicators in Pakistan from 1960 to 2018 using the autoregressive distributed lag (ARDL) modelling technique. We implemented the covariance proportion, coefficient of determination, the Durbin Watson D statistics, analysis of variance (ANOVA), variance inflating factor (VIF), the Breusch-Pagan test, the Theil’s inequality, the root mean quare error (RMSE), the mean absolute percentage error (MAPE), and the mean absolute error (MAE) for the diagnostics, efficiency, and validity of our model. Our results showed a significant association between increased CO_2_ emissions and increased electricity production from oil, gas, and other sources. An increase in electricity production from coal resources was seen to have resulted in a decrease in CO_2_ emissions. We observed that an increase in the gross domestic product (GDP) and population growth significantly contributed to the increased CO_2_ emissions. The increment in CO_2_ emissions resulting from industrial growth was not significant. The increment in CO_2_ emissions in the contemporary year is significantly associated with the preceding year’s increase. The rate of increase was very alarming, a sign that no serious efforts have been channelled in this regard to reduce this phenomenon. We call for policy dialogue to devise energy-saving and CO_2_ emission reduction strategies to minimize the impact of climate change on industrialization, population growth, and GDP growth without deterring economic and human growth. Electricity production from different sources with no or minimal CO_2_ emissions should be adopted. We also recommend rigorous tree planting nationwide to help reduce the concentration of CO_2_ in the atmosphere as well as environmental pollution.

## Introduction

Monetary development through industrialization is usually connected with negative financial externalities like air contamination [1]. Industrialization in most nonindustrial nations is frequently connected with elevated degrees of ignition of petroleum products, which brings about discharge of gases like sulphur (IV)oxide (SO_2_), nitrous oxide, carbon (II)oxide (CO), and carbon (IV)oxide (CO_2_) [2]. These standard contaminations have direct effects on human well-being and the environment. Very few studies have examined the nexus between discharge, monetary development, and energy utilization in the past [3, 4].

In recent times, there has been a critical consideration of the connection allying energy utilization and financial development with few examinations zeroing in on breaking down the connection between energy utilization and CO_2_ outflow volumes [5, 6]. In some of China’s provinces, Zhanga and Cheng [7] explored the association between energy utilization, fossil fuel by-products, and monetary development. They concluded that there is a Granger casualty unidirectional linking gross domestic product (GDP) and utilization of energy. They further revealed a Granger casualty unidirectional linking utilization of energy and CO_2_ outflow over a long haul [7].

In a comparative report, [8] saw that the utilization of energy and monetary development is connected with astronomical air contamination in a few African nations over the long haul. The connection between natural air quality and monetary development has mostly been displayed utilizing models of econometrics having the Kuznets environmental curve (KEC) as the predominant component [9–12].

The validity of the KEC has been analysed by numerous studies but with mixed findings [13]. Proof for the KEC is manifested as an altered u-shape observed in a few exact examinations [14]. Several studies have challenged the legitimacy of the KEC results [15], while most have argued that the connection between financial development and climate change can be portrayed by n-formed or u-moulded, or several forms [16]. While most studies affirmed the presence of a linkage, numerous others questioned the presence of this connection [17, 18].

Monotonic curves have been used to portray the circumstances attributed to natural contamination increments with expanded pay levels as well as the other way around [10]. However, those curves that are not monotonic have been used to depict several intricate patterns. Altered u-and n-moulded bends are among the most generally utilized non-monotonic curves [11]. Reversed u-moulded curves that are non-monotonic have stayed famous because of their effortlessness and hypothetical ability to portray the positive flexibility between ecological contamination and income levels [12]. The transformed u-curves can likewise make sense of the effects of primary changes on contamination and utilization, and effectively depict the impacts of involving green innovation on the climate as pay increments [10]. Then again, the n-formed curves indicated that the decrease in ecological contamination because of expanding income levels might be impermanent, as natural contamination deteriorates again with expanding income per capita because of expanded material utilization [19].

Proofs of the connection between gas emanations and monetary development have been found generally with nitrous oxides, and somewhat CO_2_ comes about because of the burning of petroleum derivatives [20]. While certain examinations approving the Kuznets speculation dominated automated Asia and Europe, nothing seems to have been finalized in Sub-Saharan Africa [21]. Specifically, not so many examinations have been finalized in Africa’s southern part to associate monetary development and modern gas outflows [22]. South Africa has gone through numerous financial cycles since the pilgrim long stretches of politically sanctioned racial segregation and after its vote-based administration in the mid-1990s. Needs for energy in South Africa are principally fulfilled with power from coal-terminated plants, thereby producing large amounts of CO_2_ and SO_2_ discharges [22]. South Africa has been touted as the main ozone-depleting substance producer in Africa and positions high all around the world [22]. The connection between CO_2_ discharge and monetary development in South Africa has recently been investigated regarding energy use and financial approaches [22].

Berkun and his colleagues [23] scrutinized the connection that betwixt energy utilization and monetary development during the 1960s through to somewhere 2016, noticing a somewhat opposite u-moulded connection between financial development and CO_2_. Utilizing the test of casualty of Granger, they confirmed the presence of a unidirectional causality between energy utilization and CO_2_ emanations [24], recommending financial development and an energy-driven economy. A comparable study zeroing in recently industrialized nations including South Africa saw financial development related to higher energy utilization and higher CO_2_ outflows [25].

Odhiambo [26] illustrated a bidirectional causality between monetary development and power utilization. Fossil fuel by-products per capita expanded unendingly in most parts of the world from the 1980s to somewhere 2006 because of the foundation of coal-terminated plants to augment developing homegrown and modern power requests [22]. Emissions of CO_2_ have expanded and have more than quadrupled beginning around the 1950s, with somewhere 90% increase in discharge resulting from coal combustion [27]. Emission levels represent a danger to environmental alterations and respiratory well-being. [27] recommended that countries ought to embark on manageable and efficient power energy sources to relieve the negative ecological effects.

Energy utilization and CO_2_ outflow sped up quicker at the expense of genuine gross domestic product (GDP) per capita [22]. Satellite information is important in observing the drawn-out patterns of gas discharges because of the modern burning of petroleum products [28]. A significant number of studies have utilized satellite information to connect modern emissions with financial development [29]. Exploration about the presence of KEC involving a series of satellite information for PM 2.5 discharges surrounding Beijing-Tianjin-Hebei, China, has been conducted [30, 31]. The connection between financial development and CO_2_ emissions in China has been analysed using a variety of approaches [32–36].

Caporale et al. [37] through methods of fractional integration and cointegration in a univariate framework established a long-run connection between China’s GDP growth and CO_2_ emissions. They emphasised that this long-run connection reaches an equilibrium when the factors are differenced ones. Torun et al. [38] with the application of wavelet-based technique showed that CO_2_ emissions and GDP growth have a positive interrupted short-term and strong unidirectional long-run relationship, thereby, specifying that economic growth propels environmental destruction. Wu et al. [39] used the index of logarithmic mean divisia with the decoupling Tapio model to establish that CO_2_ emissions peaks take into account air quality, path, and time in the transportation sector. They showed that the improved transportation sector fuelled CO_2_ emissions to its peak.

Onofrei et al. [40] employed cointegration analysis to study the links that betwixt CO_2_ emissions and economic growth in several European countries. In addition to the cointegration, they applied the dynamic ordinary least squares to establish a long-run alliance linking CO_2_ emissions and economic growth in Europe. They concluded that a percentage increase in GDP growth leads to a significant increase in CO_2_ emissions. Mitić et al. [41] applied panel analysis techniques to formulate a short-term bidirectional relationship linking consumption of energy, employment, and CO_2_ emissions. They further established a casual long-run interrelation linking GDP, CO_2_ emissions, and employment. They utilized the analysis of variance decomposition to contrast the existing relationships. Tong et al. [42] used bootstrap approach to autoregressive distributed lag with systematic breaks to show the casualty and cointegration linking CO_2_ emissions, economic growth, and consumption of energy in all E7 countries [43]. They indicated that there existed no cointegration between the variables in three of these countries while there existed one between the remaining four countries. It was evident from their study that CO_2_ emission are greatly influenced by energy consumption.

Using quantile regression approaches, Zhou et al. [44], we showed that CO_2_ emissions increase with the consumption of energy when unobserved heterogeneities are considered. They revealed through their analysis that CO_2_ emissions in developed countries are much greater than those in developing and less developed countries.

Alganthiran and Anaba [45] studied the effects of economic growth on CO_2_ emissions in some African countries using linear models and linear mixed models with the application of robust statistical estimation. They established that economic growth culminates in increased CO_2_ emissions by a marginal percentage. They concluded that energy consumption from oil and so on contaminated the air. Oteng-Abayie et al. [46] observed that increased economic growth activities in Ghana increased CO_2_ emissions in the country between 1990 and 2018. They applied decomposition analysis to synthesis the links that betwixt economic growth and CO_2_ emissions. They showed that there existed a weak decoupling status over the period between the two variables in the country.

The mixed technique of the interrelation linking CO_2_ emissions and economic factors has heightened the attention of policy makers, scholars, politicians, ecologists, and economists to their subsequent contribution to environmental pollution and its related issues. The continuous unlimited use of natural resources will always impact the economic development of countries [47]. This ecological disaster, specifically CO_2_ emissions, has also impacted the economic and social development goals [48]. The impact of CO_2_ emissions and its damaging effects on the environment has been examined by [49–51]. The existing universal interrelation that betwixt economic growth and carbon dioxide emissions has been validated by [52, 53]. The relationship between economic growth and CO_2_ emissions has been explored with panel data analysis in ten countries [54]. Keeping in view the significance of the connection between economic development and CO_2_ emissions will aid boasting energy supremacy policies and growth in renewable resources [55, 56].

Several studies in Pakistan have discussed the relationship between CO_2_ emissions and economic progress, energy usage, electricity consumption, human capital reduction, and non-renewable energy, but they could not establish the interaction of these factors on one another [57–64]. Pakistan is a developing country with somewhat steady economic stability. However, the advancement in the growth of the population coupled with its reliability of energy sources through coal and oil continues to fuel environmental pollution [60]. An increase in economic, industrial, and population growth was observed to have resulted in environmental degradation in Pakistan [58]. The utilization of conventional energy production through the burning of carbon was seen to have had a negative impact on the environment [65].

Pakistan is envisaged to have high CO_2_ emissions in recent years [60]. As a result, CO_2_ emissions are gaining much importance due the present environmental and demographic conditions in the country. These environmental and demographic conditions are believed to have an association with several factors that are considered in our study [59]. We are of the view that if these conditions are not addressed with the needed seriousness and concern, it may trigger a serious climate change challenge in the country, thereby affecting all areas of the economy. In this study, we estimate the nexus between CO_2_ emissions and economic determinants in Pakistan using the autoregressive distributed lag (ADL) technique. The study is unique due to it data coverage and incorporation of major economic indicators of the country as well as population growth and industrialization which are known to influence CO_2_ emissions. Thus, our study establishes the real relationship between CO_2_ emissions and economic, demographic, industrial, and energy determinants to assist the government and its stakeholders with policy directions to help curb the effect of climate change on the economy.

The next sections of the paper present the characteristics of the data and methods used together with the results and its associated discussions as well as the conclusions, recommendations, and policy implications based on the findings.

## Materials and methods

### Data

**Fig 1:**
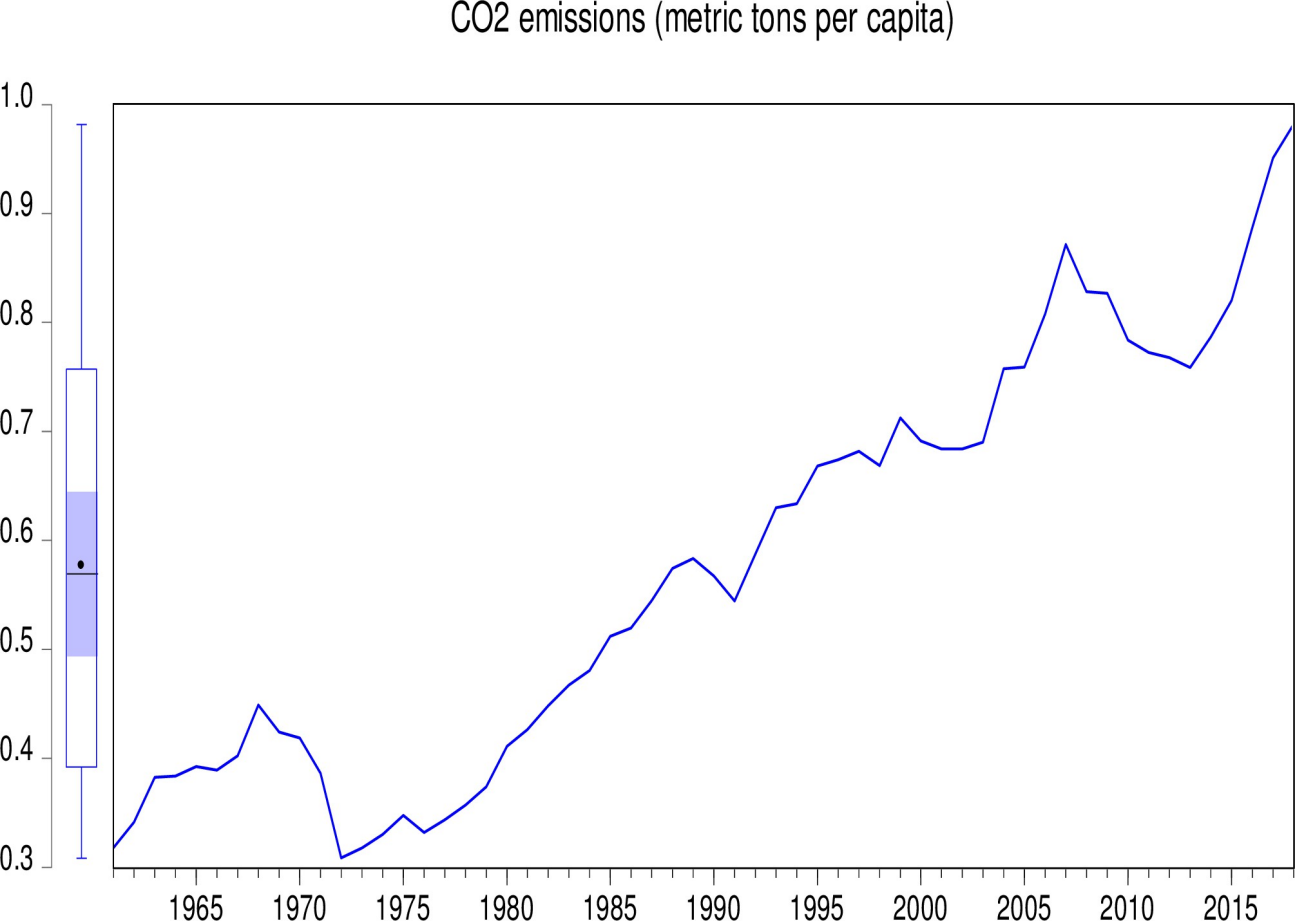
Trend of CO_2_ emissions from 1960 to 2018.

**Fig 2:**
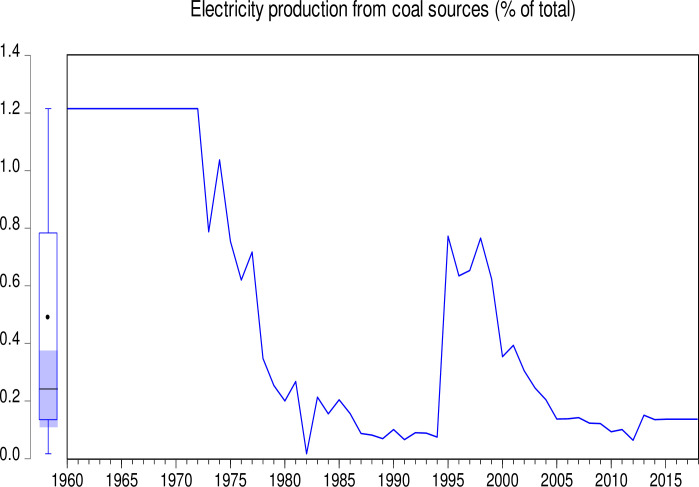
Trend of electricity production from coal sources from 1960 to 2018.

**Fig 3:**
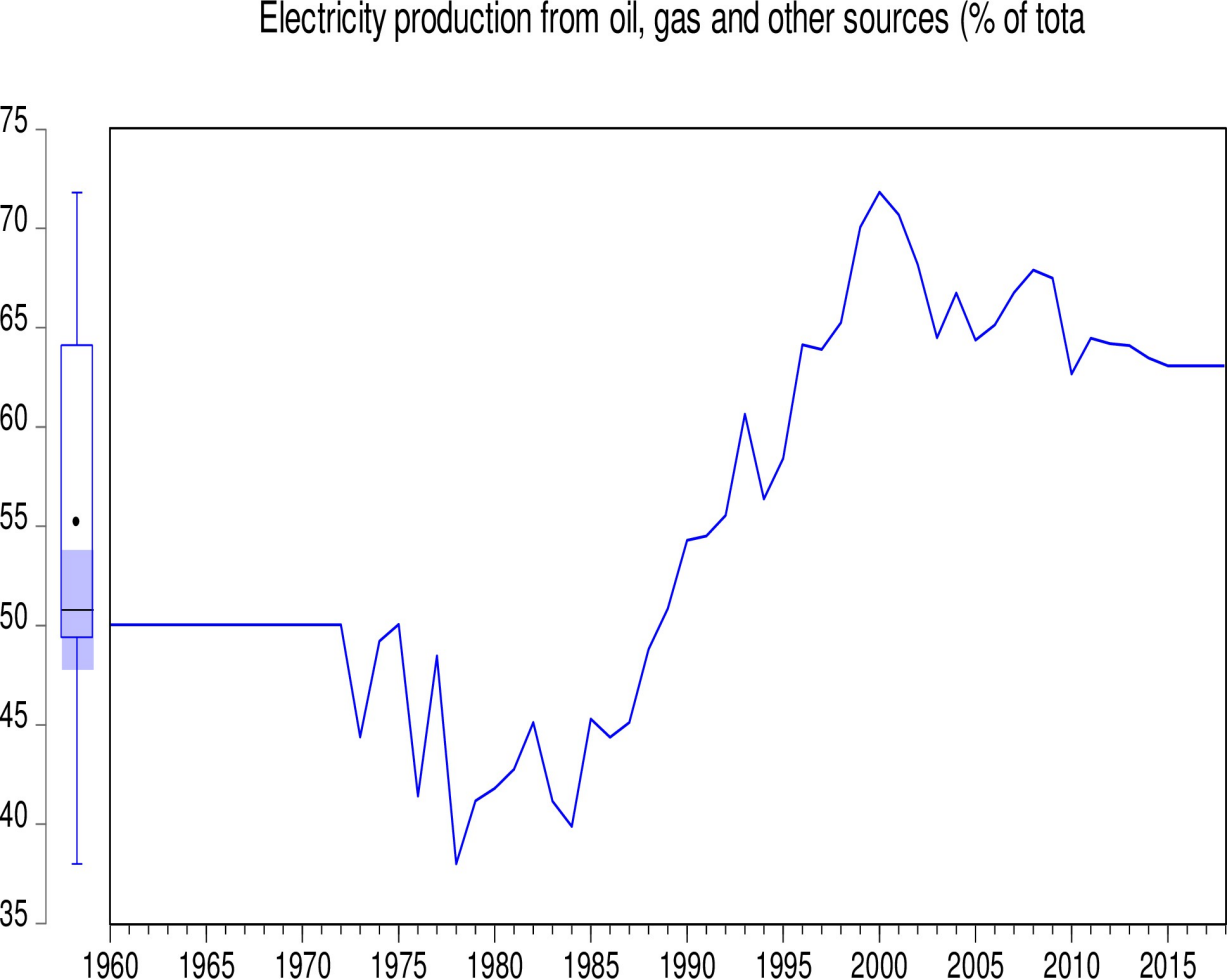
Trend of electricity production from coal sources from 1960 to 2018.

**Fig 4:**
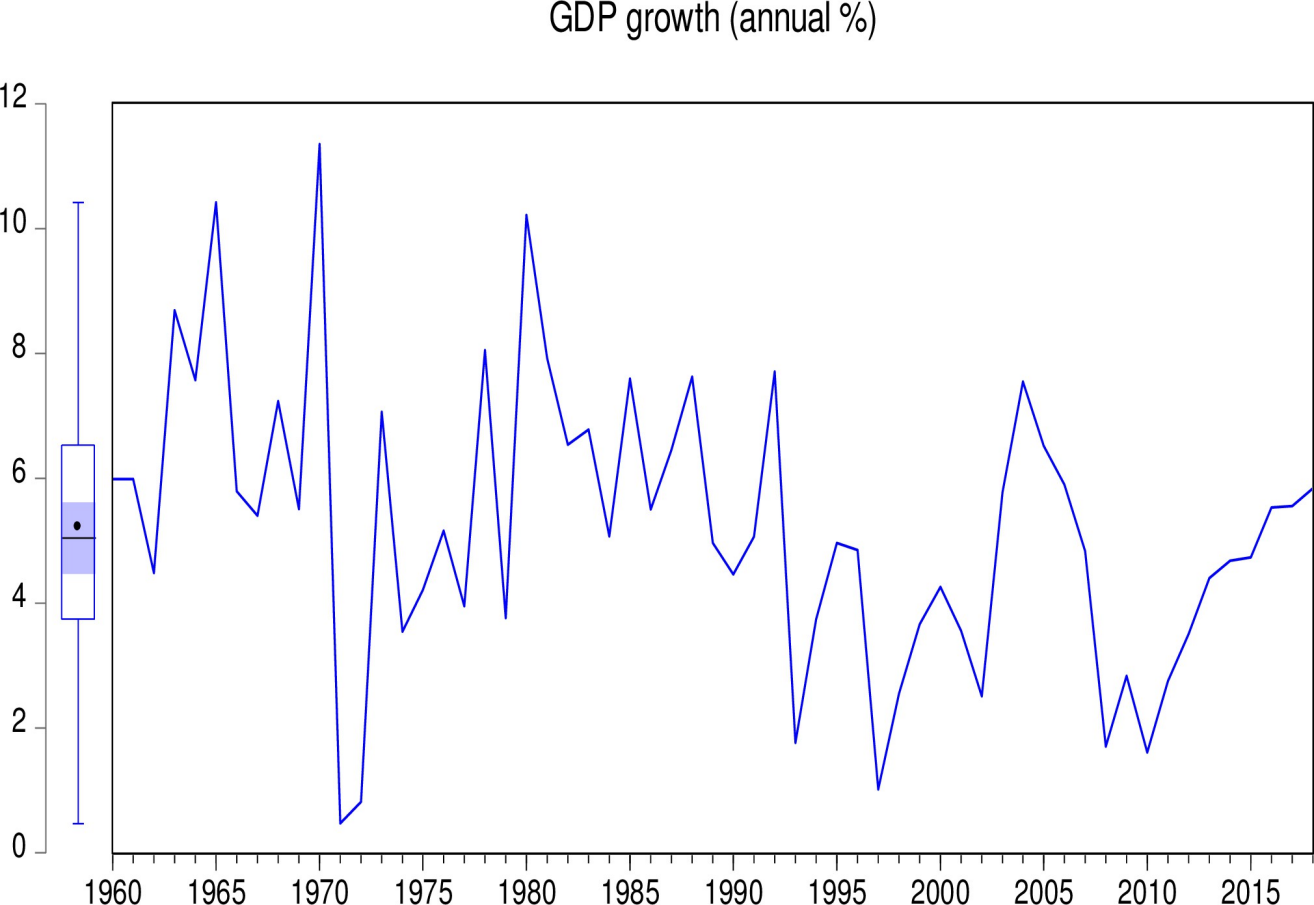
Trend of GDP growth from 1960 to 2018.

**Fig 5:**
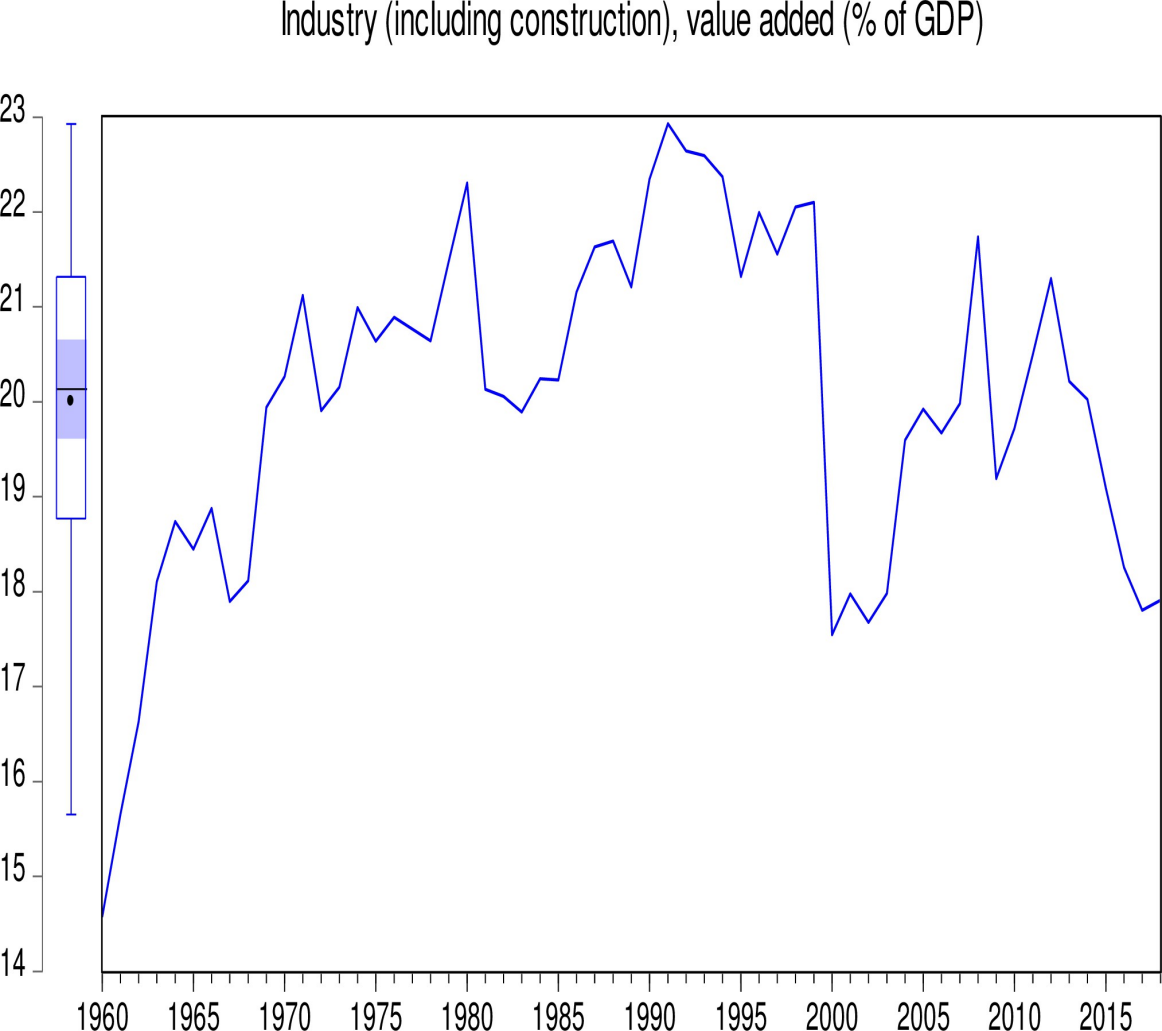
Trend of Industry growth from 1960 to 2018.

**Fig 6:**
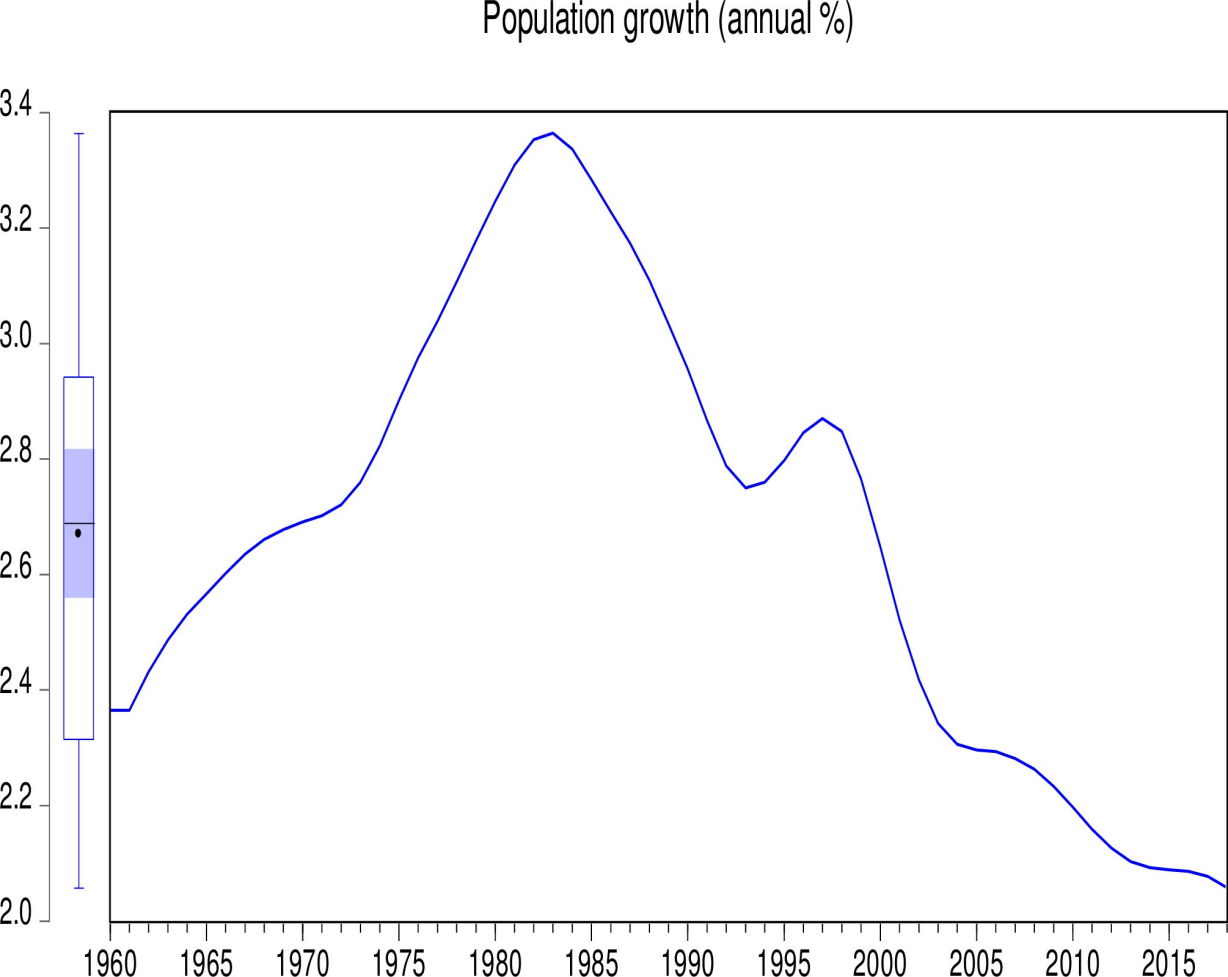
Trend of population growth from 1960 to 2018.

The data used in this study consist of CO_2_ emissions (CEs) (metric tons per capita), GDP (annual growth), population growth (PGR) (annual %), Industry (including construction) value added (IVG) (% of GDP), electricity production from coal sources (EPCS) (% of total), and electricity production from oil, gas and other sources (EPOGS) (% of total) in Pakistan from 1960 to 2018. The data was obtained from the World Bank data indicators of Pakistan (https://databank.worldbank.org/country/PAK/556d8fa6/Popular_countries#selectedDimension_WDI_Ctry). Descriptive statistics of the data is presented in Table 1. Figs 1 to 6 illustrate the graphical trends of the economic variables and CO_2_ emissions from the year 1960 to 2018.

**Table 1:** Descriptive statistics of the variables.

Variables of the Study	Minimum	Maximum	Mean	Std. Deviation	Skewness
**CEs**	0.31	0.98	0.57	0.19	0.27
**PGR**	2.06	3.36	2.67	0.39	0.11
**GDP**	0.47	11.35	5.22	2.31	0.26
**IVG**	14.57	22.93	20.01	1.79	-0.70
**EPCS**	0.02	1.22	0.49	0.45	0.72
**EPOGS**	38.00	71.83	55.19	9.44	0.04

We can observe in Fig 1 that CO_2_ emissions increased slowly from 1960 to 1968 before reducing at the same pace in 1972. However, the emissions increased astronomically after 1972. In Fig 2, the electricity production from coal sources was high in 1960 and remained constant from 1975, after which there was a general decline to 1995. A sharp increase was again observed in electricity production from coal sources from 1995 to 2000. Although this increase was not up to the 1960 level, it generally decreased thereafter. In Fig 3, electricity production from oil, gas, and other sources remained constant from 1960 to 1970, generally fluctuating in a decreasing manner to 1978. It generally increased significantly to 2001 with a slow decrease thereafter. As shown in Fig 4, Pakistan’s GDP growth rate has been sinusoidal from 1960 to 2018. The government and its stakeholders must put in place the relevant policies to stabilize the GDP growth rate into a promising one. Industry growth in the country, as illustrated in Fig 5, has been very erratic with a general increase from 1960 to 1993, which generally decreased thereafter. The annual population growth rate increased tremendously from 1960 to 1985, after which it decreased at the same pace. The rate of decline in the growth of the population, as shown in Fig 6, is very alarming. This must be further investigated with greater efforts to change the trend [66].

## Methods

### Autoregressive distributed lag model

The autoregressive distributed lag (ARDL) model has been appraised as the most appropriate econometric model in relation to others, especially in situations where the variables are deemed stationary at *I*(0) or integrated of order *I*(1) [42]. The ARDL approach is accurate for bringing out short-run and long-run adaptabilities at the same time for a small sample size by following the ordinary least squares (OLS) technique for cointegration connecting variables. The ARDL affords flexibility in the order of integration of the variables in a way by incorporating a suitable independent variable in the models with *I*(0) and *I*(1) [36, 67, 68]. The equation for the estimation of our parameters from the ARDL [69, 70] can be expressed in the following form

$$CO_{2}=a_{0}+a_{1}EPCS+a_{2}EPOGS+a_{3}GDP+a_{4}PGR+a_{1}IVG+e_{t}$$ (1)

The above expression can also be expressed in the following form;

$$\Delta Co_{2}=a_{0}+a_{1}\sum_{k=1}^{n}\Delta EPCS+a_{2}\sum_{k=1}^{n}\Delta EPOGS+a_{3}\sum_{k=1}^{n}\Delta GDP+a_{4}PGR+a_{1}\sum_{k=1}^{n}\Delta IVG+e_{t}$$ (2)

### Cointegration analysis

Before applying the ARDL model, we performed cointegration analysis to validate the existence of cointegration between CO_2_ emissions, EPCS, EPOGS, PGR, IVG, and GDP growth.

We employed the covariance proportion, the coefficient of determination, the Durbin Watson D statistics, the analysis of variance (ANOVA), the variance inflating factor (VIF), the Breusch-Pagan test, and Theil’s inequality [36, 68] to check the consistency, efficiency, and validity of our model and the inclusion of all variables.

For analyzing the performance of the forecasting model, we used the following performance measures: mean absolute error (MAE), root mean square error (RMSE), and mean absolute percentage error (MAPE) [71]. These measures can be explained as follows; here *y*_*t*_ is the observed values and *ŷ*_*t*_ are the estimated or predicted values.

The MAE is the average of all absolute errors. The formula is expressed as follows;

$$MAE=\frac{1}{N}\sum_{t=1}^{N}\left|y_{t}-{\hat{y}}_{t}\right|$$ (3)

RMSE measures the difference between observed and predicted values and is calculated using the formula

$$RMSE=\sqrt{\frac{1}{N}\sum_{t=1}^{N}{\left(y_{t}-{\hat{y}}_{t}\right)}^{2}}$$ (4)

MAPE is the mean or average of the absolute percentage errors of the forecast. The error is defined as the actual or observed value minus the forecasted value. Percentage errors are summed without regard to sign. It is expressed by the following formula

$$MAPE=\frac{1}{N}\sum_{t=1}^{N}\left|\frac{y_{t}-{\hat{y}}_{t}}{y_{t}}\right|*100$$ (5)

## Results and discussions

Pearson’s correlation coefficient that betwixt the variables together with the significance exhibited by their corresponding p-values compared with the level of significance of 0.05 is shown in Table 2. There was a moderate negative correlation between CO_2_ emissions and population growth (r = -0.59, p-value < 0.05), which means that higher CO_2_ emission relatively reduces the population growth rate. We observed a weak negative correlation between CO_2_ emissions and GDP growth rate (r = -0.28, p-value < 0.05), indicating that higher CO_2_ emission slowly reduces the GDP growth rate [72]. Although not significant, a very weak positive correlation linked CO_2_ emissions and industry growth (r = 0. 08, p-value > 0.05). This shows that Pakistan’s industry growth does not affect CO_2_ emissions. Electricity production from coal sources had a high negative correlation with CO_2_ emissions. These findings relate to the results of [53, 57].

**Table 2:** Pearson’s correlation of CO_2_ and the economic variables of the study.

Correlation	CEs	PGR	GDP	IVG	EPGS	EPOGS
**CEs**	1.00	-0.59^***^	-.028^***^	0.05	-0.64^***^	0.82^***^
**PGR**	-0.59^**^	1.00	0.21	0.47^**^	-0.01	-0.71^***^
**GDP**	-.028^*^	0.21	1.00	-0.16	0.11	-0.44^***^
**IVG**	0.05	0.47^***^	-0.16	1.00	-.39^***^	-0.08
**EPGS**	-0.64^***^	-.010	0.11	-0.397^***^	1.00	-0.29^***^
**EPOGS**	0.82^***^	-0.71^***^	-0.44^***^	-0.08	-0.29^***^	1.00

*** significant p-value < 0.05

Table 3 provides the results of the augmented Dickey Fuller test used to ascertain the stationarity of the series [73, 74]. All series were not stationary at their basic level. As a result, we differenced each series once to make it stationary at level 1 at a significant level of 0.05.

**Table 3:** ADF test for checking the stationarity of series.

Variables	Test-statistic	Pr (>│z│)
**CEs**	-5.8861	<0.0001
**EPCS**	-9.87709	<0.0001
**EPOGS**	-10.91702	<0.0001
**GDP**	-8.916967	<0.0001
**IVG**	-8.071591	<0.0001
**PGR**	-7.55678	<0.0021

We proceeded by verifying if our models’ residuals are serially not correlated with the application of the Breusch-Godfrey serial correlation test. The hypothesis for the test is

$$H_{o}:the\;residuals\;are\;uncorrelated$$

$$H_{1}:the\;residuals\;are\;correlated.$$

Our F-statistic’s p-value of 0.7828 indicates we do not have enough justification to reject the *H*_*o*_ at 0.05 significance level. We, therefore, presume that the residuals are serially uncorrelated.

To establish that there exists no multicollinearity between the independent variables, the variance inflating factor (VIF) was applied [75, 76]. From Table 4, the VIF is either close to 1 or 0, which shows that the independent variables of our study are uncorrelated indicating no multicollinearities exists between them.

**Table 4:** VIF test for independent variables.

Variables	Variance inflating factor
PGR	1.008
IVG	1.007
EPCS	0.095
EPOGS	1.002
GDP	1.004

With our significant level set at 0.05, Table 5 shows that our model gives better results at lag 0 and lag 1 compared to 2 and higher orders. With a p-value less than 0.05 for all our series under contemplation, we reject the null hypothesis of the unit root test implemented on the first difference at 0.05 significance level, thereby, concluding that no unit roots exist in the first difference. As a result, all our series are either I(0) or I(1). These results are similar to those obtained by [58–60].

**Table 5:** Cointegration analysis using intermediate ADF test.

Variables	Pr (>│z│)	Lag	Max Lag	Observations
**D(CEs)**	<0.001	0	10	57
**D(EPCS)**	< 0.001	0	10	57
**D(EPOGS)**	<0.001	0	10	57
**D(GDP)**	<0.001	1	10	56
**D(IVG)**	<0.001	0	10	57
**D(PGR)**	0.010	1	10	55

With our significant level set at 0.05, Table 6 shows that CEs has a positive significant short-run association with PGR. This means that a one percent (1%) increase in the PGR increases CEs by 8.7496 metric tons per capita. An increase of one percent (1%) in EPCS decreases CEs by 1.4782 metric tons per capita. However, increasing EPOGS by one percent (1%) significantly increases CEs by 0.1734 metric tons per capita. GDP growth rate was positively associated with CEs, increasing it by 0.2689 metric tons per capita for every one percent (1%) increase in growth. Our findings confirm the results of [37, 38, 40].

**Table 6:** Short-run estimation of the parameters of ARDL with CEs as a dependent variable.

Variable	Coefficient	Std. Error	t-Statistic	Pr (>│z│)
**Intercept**	1.0397	3.7207	0.2794	0.0781
**PGR**	8.7496	0.8054	10.8625	<0.0001
**IVG**	-0.1779	0.4050	-0.4393	0.0662
**EPGS**	-1.4782	0.4050	-0.4393	0.0600
**EPOGS**	0.1734	2.80504	-0.5270	0.0045
**GDP**	0.2689	0.5839	0.4604	0.6470

To establish the long-run interrelations that betwixt the variables, we used the ARDL bound test for the confirmation of cointegration hypothesis

$$H_{o}:there\;is\;no\;long\;–run\;relationship\;between\;the\;dependent\;and\;indepedent\;variables$$

$$H_{1}:\text{:}\;there\;is\;a\;long–run\;relationship\;between\;the\;dependent\;and\;indepedent\;variables.$$

The estimated results in Table 7 show that the value of the F-statistic, 5.72, is greater than the lower and upper bounds at 1%, 2.5%, 5%, and 10% levels of significance when EPCS, EPOGS, PGR, IVR, and GDP growth are considered as independent variables. We, therefore, do not have enough justification to reject *H*_*o*_ and presume that the ARDL bound test approves the existence of a long-term relationship between CO_2_ emissions and all independent variables. After confirming the occurrence of the long-term association by the ARDL test, we computed the parameters for the long-term associations. This result relates to the findings of [39, 40, 45].

**Table 7:** F-Bound test for the decision of long-run estimation.

F-Bounds Test				
Test Statistic	Value	Significance level	Lower bound I(0)	Upper bound I(1)
			Asymptotic: n = 1000	
**F-statistic**	5.72	**10%**	2.08	3
**K**		**5%**	2.39	3.38
		**2.5%**	2.7	3.73
		**1%**	3.06	4.15

At a significant level of 0.05, the results of the long-run ARDL in Table 8 show that CEs has a positive significant association with the previous year’s CEs rate, meaning an increase of one percent (1%) in CEs in the previous year will significantly increase the average CEs of the current year by 0.947 metric tons per capita. An increase of one percent (1%) EPCS significantly decreases CEs by 0.043 metric tons per capita. However, increasing EPOGS by one percent (1%) significantly increases CEs by 0.005 metric tons per capita. GDP growth rate increases CEs by 0.006 metric tons per capita for every one percent (1%) increase in growth. Although not significant, an increase in IVR by one percent (1%) increases CEs by 0.004 metric tons per capita. Moreover, a one percent (1%) increase in PGR rate significantly increases CO_2_ emissions by 0.241 metric tons per capita. Our results confirm the findings of [40, 61–64].

**Table 8:** Long-run estimation of the parameters of ARDL with CEs as a dependent variable.

Variable	Coefficient	Std. Error	t-Statistic	Pr (>│z│)
**CEs (-1)**	0.947	0.055	16.90	<0.0001
**EPCS**	-0.043	0.013	-3.12	0.0030
**EPOGS**	0.005	0.001	3.85	<0.0001
**EPOGS (-1)**	-0.003	0.001	-2.71	0.0090
**GDP**	0.006	0.001	4.18	<0.0001
**GDP (-1)**	0.000	0.001	0.48	0.6280
**GDP (-2)**	-0.001	0.001	-1.18	0.2420
**GDP (-3)**	0.003	0.001	2.11	0.0390
**IVP**	0.004	0.002	-1.63	0.1090
**PGR**	0.241	0.0867	2.78	0.0070
**PGR (-1)**	-0.239	0.0810	-2.95	0.0050

The model’s R-squared value of 98% shows that the model fits well. The Durbin Watson D statistic, estimated to be 1.78 (close to 2), confirms that there is no autocorrelation in our model. In the ANOVA, we observed the F statistic to be 355.268 (p < 0.0001), an attestation that the model is significant. The Breusch-Pagan test yielded a p-value of 0.3521, showing that no homoscedasticity is present in our model. The RMSE, MAE, and MAPE values were 0.03, 0.02, and 4.79, respectively. Theil’s inequality was estimated to be 0.02, which is close to 0, indicating the validity of the estimated model with a high covariance proportion of 0.95, showing the strength of the model.

## Conclusion

The study examined the nexus that betwixt CEs and its associated economic indicators in Pakistan with data from 1960 to 2018 using the ARDL modelling technique. The economic indicators include EPCS, EPOGS, PGR, IVR, and GDP growth. We implemented the covariance proportion, the coefficient of determination, the Durbin Watson D statistic, the ANOVA, the VIF, the Breusch-Pagan test, the Theil’s inequality, the RMSE, the MAPE, and the MAE for the diagnostics, efficiency, and validity of our model. The empirical results from the ARDL bound test confirmed a long-term linkage between the variables. We found a significant positive link between CEs and EPOGS. However, a negative association was observed between EPCS and CEs. We observed that PGR has a direct impact on the emissions of CO_2_. On the other hand, IVG had no significant impact on CEs. An increase in GDP growth increases CEs in the long-run. CEs from 1972 to 2018 was seen to be on the rise.

Pakistan’s economy has not doubled per capita income, however, CO_2_ emissions per capita have doubled [61]. The average economic growth stands at more than 6%, while in the 1990s, it was below 4%, whereas GDP growth was around 4% from 2000 to 2010 [62]. The government should establish hydro projects and construct eco-friendly energy-related projects with renewable energy sources [63, 64]. The Pakistan government should embark on responsible electricity production from coal sources. Presently, Pakistan is producing around 431MW of solar energy, and very little interest has been developed in producing electricity from wind energy [62]. We suggest that the importation of items that encourage eco-friendly (green) technologies should be given minimum tariffs and tax exemptions [64]. Electricity production from different energy sources (i.e., renewable and alternative) that have the ability to reduce CO_2_ emissions should be encouraged [62]. Likewise, incentives should be provided to boast research and development in the industrial and agricultural sectors so that companies become more efficient in their production and energy use.

Moreover, the government should encourage industries to import and export eco-friendly technologies and provide them tax exemptions that follow these rules [63, 64]. A nationwide awareness programme on planting more trees in all areas should be the goal of the government. Clean environmental products like Photo-voltaic solar cells, wind power generating sets, and hydro turbines should be made duty-free and subsidized for all [63, 64]. The government should provide the private and agriculture-based industries in the energy sector with special incentives and tax exemptions, etc. in order to encourage them to produce energy through minimal carbon emission techniques.
